# Supplementary material for: Novel Insights into the Mechanism Underlying High Polysaccharide Yield in Submerged Culture of Ganoderma lucidum Revealed by Transcriptome and Proteome Analyses
Source: Microorganisms. 2023 Mar 17;11(3):772. doi: 10.3390/microorganisms11030772 (PMC10055881; doi:10.3390/microorganisms11030772)
Supplement: Supplementary file 1 [file microorganisms-11-00772-s001.zip › microorganisms-2186896-supplementary.pdf]

**Supplementary Material**

*1. Proteome analyses*

After culture 8 days, the mycelia with three biological replicates in glucose and xylose group were collected for studying the total proteome. Sample was ground individually in liquid nitrogen and lysed with lysis buffer (50mM Tris-HCl, 8M Urea, 1% SDS, pH 8), followed by 5 min of ultrasonication on ice. This lysate was centrifuged at 13,000 rpm/min for 20 min at 4 ℃, and the supernatant was added four volumes of precooled acetone with a final concentration of 10 mM dithiothreitol and incubated 2 hours. Samples were then centrifuged and the precipitation digested by adding 1.5 μg sequencing grade trypsin (Promega, USA) and 500 μL of 100 mM TEAB buffer, and incubated at 37 °C for 4 hours. And then, 1.5 μg trypsin and $CaCl_2$ were added and incubated for overnight at 37 °C. Resulting peptides were purified three times with C18 desalting column with washing buffer (0.1% formic acid, 3% acetonitrile), and redissolved in elution buffer (0.1% formic acid, 70% acetonitrile). The eluents of each sample were combined and lyophilized.

The separated peptides were analyzed by EASY-nLC™ 1200 UPLC connected to a Q Exactive HF mass spectrometer (Thermo Fisher Scientific, United States), with ion source of Nanospray Flex™. Each sample was separated using a two-column set-up consisting of a C18 Nano-Trap column (2 cm×75 μm, 3 μm) and an analytical column (15 cm×150 μm, 1.9 μm). The linear separation gradient consisted two solvent systems (buffer A: 100% water and 0.1% formic acid; buffer B: 80% acetonitrile and 0.1% formic acid) for 60 min at a flow rate of 600 nL/min. Full scan range from m/z 350 to 1500 with resolution of 60000. The top 40 precursors of the highest abundant in the full scan were selected and fragmented by higher energy collisional dissociation.

MS/MS spectra were screened by Proteome DiscovererTM 2.2 software. Error tolerances on the precursor and fragment ions were ± 10 ppm and ± 0.02 Da, respectively. Carbamidomethyl was specified as fixed modifications, Oxidation of methionine (M) was specified as dynamic modification, and acetylation was specified as N-Terminal modification. A maximum of 2 missed cleavage sites were allowed. The identified Peptide Spectrum Matches (PSMs) and protein were retained and performed with FDR no more than 1.0%. The protein quantitation results were statistically analyzed by T-test. Differential expression analysis on the

proteins identified with $p$-value < 0.05 and |Log$_2$(Fold Change)| > 1.

**Table S1** Primers used in this study

| Gene name | Primer sequence | |
|---|---|---|
| *PGM* | Forward | 5'-GGGCCTGAGGAAGAGGGTGA-3' |
| | Reverse | 5'-CGGTTTCGGGGGAGAAGTAG-3' |
| *UGP* | Forward | 5'-TGGTCTCGGAACTTCTATGGG-3' |
| | Reverse | 5'-CAGTGCTTCTTCTCGTCCTCA-3' |
| *GL22527-R1* | Forward | 5'-GCACCATTACCACCAGACGA-3' |
| | Reverse | 5'-TGGTGTTCATCCCATCACGG-3' |
| *GL20535-R1* | Forward | 5'-GTACCTCATGGACCTCGTGC-3' |
| | Reverse | 5'-CAGATCGACAGACCCAACCC-3' |
| *GL27365-R1* | Forward | 5'-CGTGAACAGATGCGGGTCTA-3' |
| | Reverse | 5'-GGTCGAGCCTTGTACCGAAA-3' |
| *GL30087-R1* | Forward | 5'-TGGCATTCCAACCAGACGAA-3' |
| | Reverse | 5'-ACAGCGGAGAGAACATTGGG-3' |

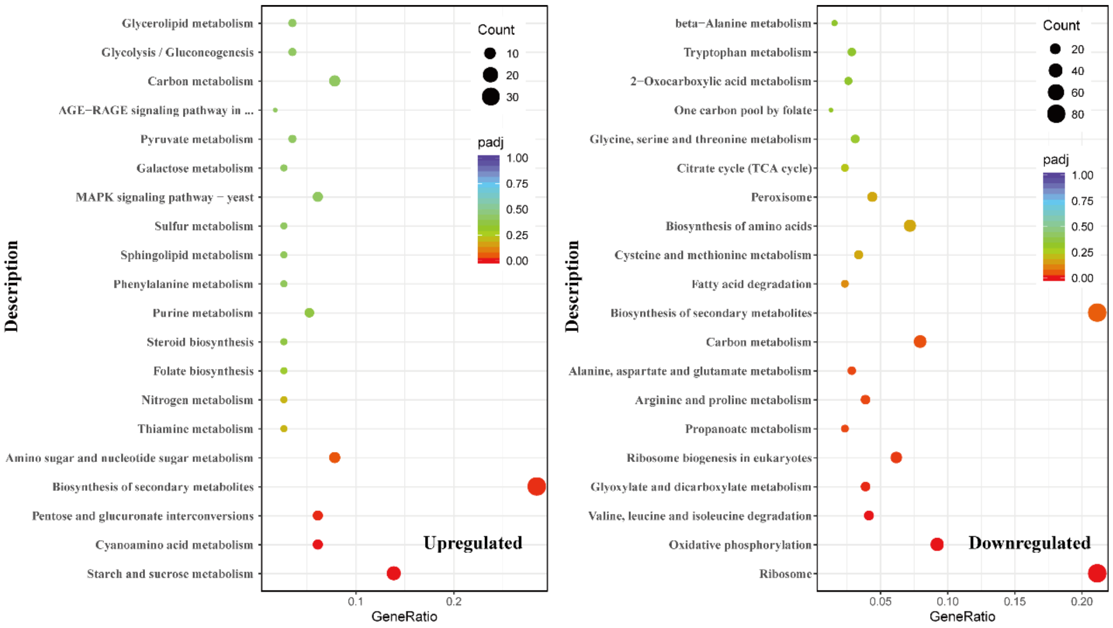


**Figure S1.** Statistics of KEGG pathway enrichment. The top 20 enriched pathways were shown. The left is the KEGG analysis of 1779 upregulated genes and the right is the KEGG analysis of 1650 downregulated genes (Glucose *vs* Xylose).
